# Assuring the Future of Basic Biomedical Science

**DOI:** 10.1093/function/zqac006

**Published:** 2022-02-26

**Authors:** Allen W Cowley

**Affiliations:** Medical College of Wisconsin, Milwaukee, WI 53226, USA

This editorial was prompted by a recent meeting of the Fellows of the International Union of Physiological Sciences (IUPS) during which ideas were solicited for how to advance physiology globally. An endeavor that I believe deserves our serious consideration is the serious engagement of patients, caregivers, and advocates into as many of our national and international activities as possible. There has been a rapidly growing distrust of science which we must recognize and respond to. This mistrust threatens our very future since why should we expect strong continued public funding of our research if they no longer trust us? Informed patients when provided meaningful opportunities are our strongest allies and we must find ways to integrate this energy into our efforts to assure the future of basic biomedical research.

As in most complex dynamic systems, biological or societal, when stressed the weaknesses become apparent. Such is the case since the onset of the Covid pandemic in 2020 which has tested every aspect of our scientific, medical care, and public health enterprises. Ironically, the pandemic has revealed a growing erosion of public confidence in science and biomedical research. This was starkly reflected in the recent population survey on confidence in institutions conducted by NORC at the University of Chicago^1^. Although about three-quarters of Americans believe that the benefits of scientific research outweigh any harmful results, only 50% express a “great deal of confidence” in the scientific community. Not a rousing endorsement. This is despite the remarkable achievements in science and medicine over these recent decades and the successful and heroic responses to the Covid pandemic. This should be distressing to us all. As Rice University historian Douglas Brinkley has said, “Science used to be something all Americans would get behind, but we now see it falling prey to the great political divide. The world of science should be a meeting house where right and left can agree on data. Instead, it's becoming a sharp razor's edge of conflict.”

Why? The reasons for this are clearly complex and not easily addressed. First, in all fields of science the work that we do is highly specialized and cloaked in a language of symbols and acronyms unfamiliar to most, including many health care professionals. Few of our scientific reports are ever read by practicing physicians or the general public. Although there are journalistic efforts to highlight basic discoveries for the non-scientists, it is evident that even these sources of information are increasingly drowned out by social media scientific hucksters and a variety of “fake news” sources. So even when exciting real scientific discoveries are made and reported in reputable news sources such as the New York Times science section, as it is disseminated the social media to the general public it is met with skepticism by at least half of our population. Efforts of our scientific societies to communicate such achievements are largely never seen by the public, most of whom have little awareness that our societies even exist.

Second, we must not forget that politicization and mistrust of science pitting fact versus fiction has been with us for centuries, as Galileo could tell us. The problem waxes and wanes but is ever present. It was not long ago that it was believed that science and technology would rid the world of starvation and diseases as reflected in Aldous Huxley's “Brave New World” and other writers of the early 20th century. These feeling prevailed throughout the period of the Cold War and propelled us to the moon and the sequencing the Human Genome by the year 2000. Yet, with the spectacular rise of technology, the social media, and the vast wealth of Silicon Valley, we have seen the depersonalization and fragmentation of our social structures. Most threatening has been the rising income inequality, health disparities placing the fruits of our scientific endeavors out of reach for an increasing number of people. The skyrocketing cost of the US health care system has placed a large portion (∼40%) of our population in a state of constant worry that they or a family member will be unable to obtain or pay for care when needed. Science and technology are viewed by many as having contributed to the high cost of care. The high cost of prescription drugs, the profligate use of expensive high tech diagnostic instrumentation when coupled with excess profiteering by our health providers and insurers has contributed to erosion of the public support of the scientific community.

Although few scientists are apathetic to these issues, there is a sense that little can be done to change things. Our usual public relations efforts by our professional societies appear to have done little to dissuade the public of their mistrust of science. At this critical juncture, it is time to try something different assuming that most of us subscribe to Einstein's adage that insanity is doing the same thing over and over and expecting a different result. Clearly there is no single solution, but I propose that a meaningful place to start would be a serious engagement of our scientists with those who stand to gain the most from our research, the patients and our community health care providers. A National Academy of Medicine study has emphasized the need to elicit and understand patient perspectives within their unique psychosocial and cultural contexts to reach a shared understanding of patient problems and treatments^2^. I would propose that the concept of “patient centeredness” should be extended into our world of science.

Patient advocacy has been demonstrated to be a powerful tool to advance scientific research and medicine. Among the most remarkable examples of this have been the efforts of Mary Lasker whose advocacy brought about the “War on Cancer” and the passage of the National Cancer Act of 1971 which ultimately led to the 21st Century Cures Act allocating and additional $6.56 billion to the National Cancer Institute and most recently the announcement of President Biden's “Cancer Moonshot”. Who can forget the fierce advocacy mounted by patients and their friends to address the AIDS pandemic the 1980s which led to the Office of AIDS Research (OAR) reaching across the Institutes of the NIH into every area of medicine and scientific investigation representing the largest public investment in HIV/AIDS research? In the face of great adversity and stigmatization, these patient advocates overcame many political and governmental barriers resulting in the rapid discoveries leading to advancements in diagnostics and novel therapies to contain these devastating diseases.

Can basic scientists and our relatively small and fragmented organizations successfully organize such efforts? I do not believe we can do this alone, but it is perhaps possible if we could indeed harness patient power. As emphasized in a United Nations Human Development Report, “People today have an urge- an impatient urge – to participate in the events and processes that shape their lives. Properly harnessed this resource can become a source of tremendous vitality and innovation”. Patients and their families are inevitably and understandably impatient with the slow progress of science related to their conditions. But as they better understand the complexity of the biological problems and the great dedication of the scientists committed to understand their conditions, their energies and frustrations can become a great source of support in ways that mutually befit scientists and patients.

While patients clearly have much to contribute, scientists in turn must respect and help these individuals understand what we are doing and how we do it. Patients must understand the efforts and resources required to achieve what may seem like such small incremental steps in our understanding. We must conversely understand and respect the real-world experiences patients and the daily lives they live with their conditions. We must appreciate the suffering and compromised quality of life of these individuals deserving no less than our fullest attention to their ultimate needs. More than lip service to patient-scientist partnerships is required. Patients, caregivers, and advocates need to be integrated into every possible aspect of our scientific lives. They represent an enormous untapped wealth of support for the science that we do. They can bring to our scientific organizations vast experience and expertise in many areas beyond science. Their voice needs to be heard through the power of social media in getting the correct scientific messages out to the public.

Scientists seldom have an opportunity to hear from the rank-and-file patients and families of their communities. It is usually by chance conversations with our immediate friends and neighbors that we hear patients describing their disorders, needs and health experiences. Formal engagement with such individuals if carried out in serious and organized ways could go a long way to build an understanding of science and scientists. The various talents and energy of these individuals could be brought to bear to inform the long-range planning activities of our scientific societies and in the planning of scientific conferences. Patients should be integrated in various ways into our scientific meetings to communicate to our scientists. They should participate in our publications. Many patients are indeed writers and even journalists who could write opinion pieces. There are many ways that they could help bridge the communication gap to our health care providers and public. They should be incorporated into the planning and development of our societal educational activities. However, they in turn need to understand how we do our work and understand the scientific discovery process so they can provide clear and meaningful scientific messaging to schools, businesses, and civic organizations, and to patient organizations. As this begins to occur, trust and public support of fundamental research and scientists will be regained.

Patient advocate organizations play significant roles in educating the public and in lobbying government officials with aims of driving legislation and research funding related to the diseases they represent^3^. Some are large organizations such as the American Heart Association and the Cancer Society, but most are small grass roots non-profit groups seeking to raise public awareness of a disease to promote research to cure or prevent the disorder. It is the small and often struggling grass roots organizations with whom I believe we could expect to achieve the most productive relationships. Many of these are run by patients or former patients with serious diseases. These individuals have credibility with the public, legislators, and government agencies (such as the NIH) and are frequently consulted. Many of these disorders represent long neglected and often stigmatized medical conditions that have fallen between the cracks of serious attention by both scientists, physicians and our funding agencies. We should work together with these organizations and harness their strong personal commitments and energies, their personal insights and indeed the valuable information they can provide regarding their disorders.

I have personally experienced the energy and vitality of one such organization, The TMJ Association (TMJA), which recently succeeded in obtaining NIH support for a National Academy of Sciences, Engineering, and Medicine study on Temporomandibular Disorders (TMJ) which covered every aspect of TMJ. The report revealed the terrible toll on affected individuals, their families of this stigmatized and poorly studied condition^4^. My experience working with these patients has been exciting, informative and motivating. It is remarkable that when the intimidating barriers of patients speaking to scientists and scientists communicating with patients are overcome much is learned by both. It is not all that difficult to put aside scientific jargon and communicate in ways that enable meaningful discussions. Patients discover that scientists are not so intimidating, and the scientist gains a deep understanding of the relevance of what they do.

Working partnerships between scientists and patients can have a profound impact on the many issues currently faced by both. It is a process of breaking traditions, overcoming preconceived notions, and the building of new bonds. I urge our scientific societies to closely embrace the patients and their advocates. They will in turn become much more than mere bystanders and observers, but a part of our scientific team. They will be our strongest allies and advocates as they develop an understanding of the mutual challenges and needs, the process, and the benefits of science. We can no longer afford to work in our ivory towers, real or virtual, surrounded by an ever-widening skepticism and hostility of science and medicine. No one has a greater stake in understanding the science of their disorders than the patients. If there was ever a time that we need the help of these highly motivated individuals to justify and assure the future of biomedical science, it is now.
